# Synergistic Action of Flavonoids, Baicalein, and Daidzein in Estrogenic and Neuroprotective Effects: A Development of Potential Health Products and Therapeutic Drugs against Alzheimer's Disease

**DOI:** 10.1155/2013/635694

**Published:** 2013-08-24

**Authors:** Roy C. Y. Choi, Judy T. T. Zhu, Amanda W. Y. Yung, Pinky S. C. Lee, Sherry L. Xu, Ava J. Y. Guo, Kevin Y. Zhu, Tina T. X. Dong, Karl W. K. Tsim

**Affiliations:** Division of Life Science and Center for Chinese Medicine, State Key Laboratory of Molecular Neuroscience, The Hong Kong University of Science and Technology, Clear Water Bay Road, Kowloon, Hong Kong

## Abstract

Despite the classical hormonal effect, estrogen has been reported to mediate neuroprotection in the brain, which leads to the searching of estrogen-like substances for treating neurodegenerative diseases. Flavonoids, a group of natural compounds, are well known to possess estrogenic effects and used to substitute estrogen, that is, phytoestrogen. Flavonoid serves as one of the potential targets for the development of natural supplements and therapeutic drugs against different diseases. The neuroprotection activity of flavonoids was chosen for a possible development of anti-Alzheimer's drugs or food supplements. The estrogenic activity of two flavonoids, baicalein and daidzein, were demonstrated by their strong abilities in stimulating estrogen receptor phosphorylation and transcriptional activation of estrogen responsive element in MCF-7 breast cells. The neuroprotection effects of flavonoids against **β**-amyloid (A**β**) were revealed by their inhibition effects on *in vitro* A**β** aggregation and A**β**-induced cytotoxicity in PC12 neuronal cells. More importantly, the estrogenic and neuroprotective activities of individual flavonoid could be further enhanced by the cotreatment in the cultures. Taken together, this synergistic effect of baicalein and daidzein might serve as a method to improve the therapeutic efficacy of different flavonoids against A**β**, which might be crucial in developing those flavonoidsin treating Alzheimer's disease in the future.

## 1. Introduction

Estrogen is one of the critical hormones for human, which possesses a wide range of physiological functions in different tissues [1]. There are three major naturally occurring estrogens in women, estradiol, estriol, and estrone. Amongst all, 17*β*-estradiol is the primary and major form of estrogen. Estrogen is mainly produced by the developing follicles in ovaries, corpus luteum, and placenta during pregnancy. After menopause, the blood level of estrogen in women is dramatically reduced, which causes a number of symptoms with different intensities. The vasomotor symptoms include hot flushes and palpitations, while the physiological symptoms include depression, anxiety, mood swings, and lack of concentration. Clinically, hormone replacement therapy (HRT) is a kind of medical treatment for treating menopausal symptoms, which artificially increases the hormonal level by a series of drugs [2]. Unfortunately, numerous findings suggest that HRT possesses various side effects that contradict to its beneficial effects in releasing menopausal symptoms. Usually, undiagnosed vaginal bleeding, endometrial cancer, venous thrombosis, and even coronary artery disease have been reported to associate with patients who received HRT [3, 4]. Interestingly, a statistical analysis revealed that the HRT-treated patients experienced a reduced risk of Alzheimer's disease [5]. This notion is further supported by the *in vitro* experiments that estrogen has neuromodulatory and neuroprotective roles in cultured neurons [6, 7]. The anatomical evidence also indicated that estrogen receptors were found in the hippocampus and cerebral cortex, in which the hormone was able to stimulate the growth of neurons [8, 9]. However, the mechanism underlying the estrogen-mediated neuroprotection has not been fully elucidated. These observations indeed suggest that estrogen may exert different biological functions in the nervous system. This raises a new functional role of estrogen regardless its classical hormonal effects and helps the developing a novel treatment against Alzheimer's disease and other neurodegenerative diseases [10]. In clinical pathology, Alzheimer's disease is manifested by different causes, including selective oxidative stress, deposition of *β*-amyloid (A*β*) peptide into senile plaques, and formation of neurofibrillary tangles. All these cellular and molecular events ultimately trigger neuronal cell death and then impair the brain functions. Therefore, different therapeutic efforts are targeted to block A*β* aggregation, A*β* production, A*β*-induced toxicity, and/or oxidative stress [11–13].

Flavonoids, a group of phytochemicals, refer to a class of plant secondary metabolites. The number of flavonoids known at the end of 2004 was reported to be 8,150. In general, the structures of flavonoids are rather diversified, but most of them share the structural similarity to estrogen [14, 15]. They can be divided into different subclasses, including: flavones, isoflavones, flavanones, flavonols, flavanonols, chalcones, dihydrochalcones, flavanes, and aurones. The average daily dose of flavonoids intake is estimated to be 20–800 mg [16]. In USA, the estimated amount of total flavonoid intake per day is 189.7 mg [17]. This high variation can be attributed to widely differing intake levels of specific food sources of flavonoids. Clinically, the side effects of estrogen in disease therapy reduce its clinical use, which provide a driving force for searching the alternative medicines of estrogen. Fortunately, the isoflavones including daidzein, genistein, and various soybean extracts, named as phytoestrogen, have been used as the alternatives for estrogen to ameliorate postmenopause symptoms. These isoflavones can be found in the commercial market. Furthermore, no adverse effect or toxicity related to phytoestrogen treatments was reported in any of the studies, including drug discovery against Alzheimer's disease [3, 18]. In addition, flavonoid-rich foods have been observed to enhance the cortical blood flow in order to improve memory and learning [19]. On the other hand, herbal medicines with high amounts of flavonoids such as Astragali Radix (Huangqi), Puerariae Radix (Gegen), and Ginkgo Semen (Yinxing) have been known to improve memory ability. As a large group of natural compounds existed in food and herbal medicines with strong antioxidative activity [20], flavonoids can be developed into an effective supplement and/or drug for the treatment of Alzheimer's disease [21]. Indeed, a variety of biological activities of flavonoids were frequently reported, including antioxidative, antiinflammatory, estrogenic, anticancer, anti-microbial, erythropoietic, osteogenic, and neuroprotective effects [22–27]. Due to the great diversity and source of flavonoids, only a small part of them have been scientifically studied, while the remaining can still provide a huge source of candidates for drug discovery.

According to our previous findings, we reported the estrogenic activities and neuroprotection activities of different flavonoids and found that some flavonoids simultaneously possessed estrogenic and/or neuroprotective effects [28–31]. This information might be useful to provide the potential candidates for the development of natural supplements and drugs against neurological diseases. In the current study, we specifically focused on the synergistic effect of two flavonoids, baicalein and daidzein, in mediating neuroprotection, and indicated the feasibility of employing this cotreatment approach to enhance the efficacy of estrogenic and neuroprotective activities of different flavonoids.

## 2. Materials and Methods

### 2.1. Cell Cultures

Human mammary epithelial carcinoma cell line MCF-7 was obtained from American Type Culture Collection (ATCC, Manassas, VA, USA) and maintained in modified Eagle's medium (MEM), supplemented with 10% fetal bovine serum, 1 mM nonessential amino acids, 0.1 mM sodium pyruvate, 100 units/mL penicillin, and 100 *μ*g/mL streptomycin in a humidified CO_2_ (5%) incubator at 37°C. Before drug treatment, cultures were washed by phosphate buffered saline (PBS), and the medium was changed to MEM-*α* (phenol red free) containing 5% charcoal dextran-treated fetal bovine serum for 2 days. Rat pheochromcytoma PC12 cell was obtained from ATCC and maintained in Dulbecco's modified Eagles' medium (DMEM) supplemented with 6% fetal bovine serum and 6% horse serum at 37°C in a water-saturated 7.5% CO_2_ incubator. All reagents for cell cultures were purchased from Invitrogen (Carlsbad, CA, USA).

### 2.2. Estrogen Promoter Activity Assay in MCF-7 Cells

The 3-repeat of estrogen responsive elements (5′-GGT CAC AGT GAC C-3′), synthesized as in [24], was subcloned into a luciferase-reporter vector pTAL-Luc (Clontech, Mountain View, CA, USA), to form pERE-Luc. Stable cell line of MCF-7 transfected with pERE-Luc was established as described previously [28]. To determine the estrogenic activity of flavonoids, the cultures were treated with 10 *μ*M estrogen (as a positive control) or different doses of flavonoids for 24 hours. Cultures were then collected by lysis buffer containing 0.2% Triton X-100, 1 mM dithiothreitol, and 100 mM potassium phosphate buffer (pH 7.8) and subjected to luciferase and protein assays. Luciferase assay was performed by a commercial kit (Tropix Inc., Bedford, MA, USA). The readings corresponding to luciferase were quantified by FLUOstar Optima (BMG Labtech, Offenburg, Germany), where the reading of luciferase activity was normalized by protein amount in each sample. 

### 2.3. Estrogen Receptor Phosphorylation in MCF-7 Cells

The amounts of phosphorylated estrogen receptor *α* (P-ER*α*) at serine 118 (S118) and total ER*α* were determined by western blotting [32]. Cultures were serum-starved for 3 hours before the drug applications. After drug treatments, cultures were collected immediately in lysis buffer (125 mM Tris-HCl, 2% SDS, 10% glycerol, 200 mM 2-mercaptoethanol, pH 6.8) and subjected to western blotting using antiphospho-estrogen receptor *α* S118 and antitotal estrogen receptor *α* (1 : 2000; Upstate, Lake Placid, NY, USA). Glyceraldehyde-3-phosphate dehydrogenase (GAPDH; 1 : 5,000; Sigma) served as an internal control for equal loading. The immunocomplexes were visualized by the enhanced chemiluminescence (ECL) method. The band intensities, recognized by the antibodies in the ECL film, in control and agonist-stimulated samples were run on the same gel and under strictly standardized ECL conditions including the amounts of primary and secondary antibodies, the incubation time for antibodies, the amount of ECL chemicals and the time for image development. The bands were compared on an image analyzer, using in each case a calibration plot constructed from a parallel gel with serial dilution of one of those samples: this was to ensure the subsaturation of the gel exposure. 

### 2.4. Determination of A*β* Aggregation and Its Cytotoxicity

A*β* (1–40) peptide at final concentration 10 *μ*M was mixed with the flavonoids and incubated for 3 days at 37°C according to the previous method [28]. Fifty *μ*L sample was then mixed with 150 *μ*L thioflavin solution (5 *μ*M in 50 mM glycine, pH 8.5) to determine A*β* aggregation by measuring the fluorescence intensity at emission 435 nm and excitation 480 nm (SPECTRA max GEMINI XS). In the A*β*-induced cytotoxicity test, cultured PC12 cells in 96-well plate were treated with A*β* (aggregated for 4 days at 37°C) for 24 hours. Followed by addition of 3-(4,5-dimethylthiazol-2)-2,5-diphenyltetrazolium bromide (MTT; Sigma) in PBS at final concentration of 0.5 mg/mL for 2 hours, the medium was aspirated, and the cultures were resuspended by 150 *μ*L DMSO to determine the cell viability by measuring the absorbance at 570 nm. 

### 2.5. Calculation of Drug-to-Drug Synergism

The multiple drug effect analysis was used to examine the drug interaction according to the median-effect principle [33–35]. This involved the plotting of dose effective curves for each drug and two drugs together in different concentrations using the median effect equation (*F*_*a*_/*F*_*u*_ = (*D*/*D*_*m*_)^*m*^ where *D* was dose; *D*_*m*_ was the dose required for 50% effect; *F*_*a*_ was the fraction effected by *D*; *F*_*u*_ was the unaffected fraction (1 − *F*_*a*_); and *m* was the coefficient of sigmoidicity of the dose-effect curve. A combination index (CI) was then determined using the classical isobologram equation of Chou-Talalay: CI = (*D*)_1_/(*Dx*)_1_ + (*D*)_2_/(*Dx*)_2_. (*Dx*)_1_ was the dose of drug 1 required to produce *x*% effect alone; (*D*)_1_ was the dose of drug 1 required to produce the same *x*% effect in combination with (*D*)_2_; (*Dx*)_2_ was the dose of drug 2 required to produce *x*% effect alone; and (*D*)_2_ was the dose of drug 1 required to produce the same *x*% effect in combination with (*D*)_1_. Values of CI referred to CI close to 1 = additive effect; CI > 1 = antagonistic effect; and CI < 1 = synergistic effect. For dose-reduction index (DRI), it provided a measure of how much the dose of each drug in a synergistic combination might be reduced at a given effect level (i.e., at *x*% inhibition) compared with the doses of each drug alone. The DRI value was calculated as (DRI)_1_ = (*Dx*)_1_/(*D*)_1_ and (DRI)_2_ = (*Dx*)_2_/(*D*)_2_ [33–35].

### 2.6. Other Assays

Protein concentrations were measured routinely by Bradford's method with a kit from Bio-Rad Laboratories (Hercules, CA, USA). Statistical tests were done by using one-way ANOVA. The data was separated into two groups to compare both the activation of drugs over the control. The control group was varied in different experiments, which was specified in the figure legends. Data are expressed as the means ± SEM of 4 independent experiments. Statistically significant changes were classed as *P < 0.05, **P < 0.01, and ***P < 0.001.

## 3. Results

**Figure 1:**
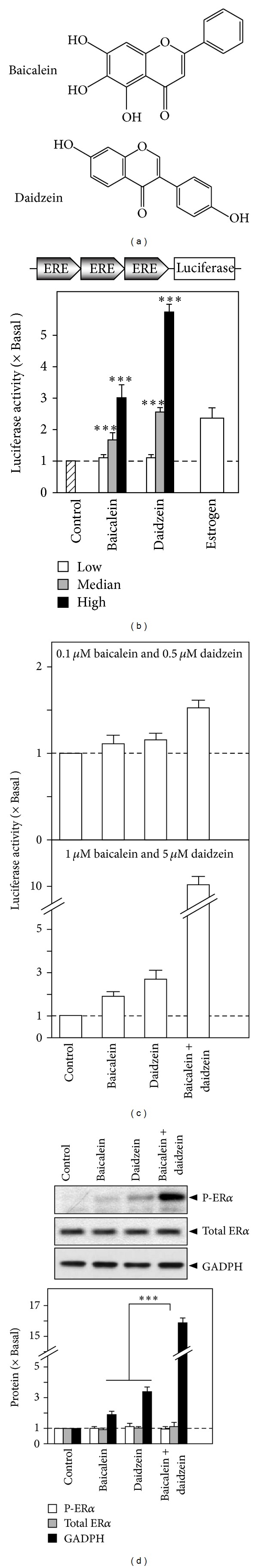
Potentiation of estrogenic activities by baicalein and daidzein. (a) The structures of baicalein and daidzein were illustrated. (b) Three repeats of estrogen responsive element (ERE) were tagged with a luciferase report gene to form pERE-Luc (upper panel). This construct was stably transfected into MCF-7 cells to determine the estrogenic activity of flavonoids. After treatment of baicalein (0.1, 1, 10 *μ*M), daidzein (0.5, 5, 50 *μ*M), and estrogen (10 nM) for 48 hours, cultures were collected to determine the luciferase activity. (c) Potentiation effect was investigated by the addition of baicalein and daidzein at 0.1 *μ*M + 5 *μ*M (upper panel) and 1 *μ*M + 5 *μ*M (lower panel) in pERE-Luc stably transfected MCF-7 cells and then performed the same assay as in (b). (d) Cultured MCF-7 cells were serum-starved and then treated with submaximal dose of baicalein (1 *μ*M) and daidzein (5 *μ*M) for 30 min. Phosphorylation of estrogen receptor *α* (P-ER*α*) was determined by western blot analysis. Total ER*α* and GADPH served as a loading control. Quantification of band intensity was performed in lower panel. Data are expressed as *x* Basal, whereas reading of control without drug or time 0 min is set as 1, mean ± SEM, *n* = 5. ***P < 0.001.

One of the cellular characteristics of flavonoids is their abilities to stimulate estrogenic response. To demonstrate this biological effect of baicalein and daidzein (Figure 1(a)), a stable MCF-7 breast cancer cells, harboring three repeats of ERE in a luciferase-reporter (pERE-Luc) corresponding to the transcriptional activity of estrogen receptor (ER), were employed (Figure 1(b)). Experimentally, pERE-Luc expressed MCF-7 cultures were treated with DMSO (mock control), baicalein, daidzein, at different concentrations for 24 hours and then collected to perform luciferase activity assay. Results showed that both baicalein and daidzein were able to stimulate the transcriptional activity of ERE in a dose-dependent manner, with maximum response up to 3-fold and 6-fold, respectively (Figure 1(b)). Estrogen (10 nM) served as a positive control for the assay. 

In general, the estrogenic activity of flavonoids can be enhanced by increasing the working concentration, as shown in our results. However, this may not be applicable in all cases due to the limitation of drug solubility and cytotoxicity. To solve this issue, the combination of different flavonoids with similar biological properties can be employed to increase their cellular efficacy, as well as to lower the possible side effects of the drugs when used in high concentration. Based on this rationale, baicalein and daidzein were mixed together at concentration of 0.1 *μ*M + 0.5 *μ*M and 1 *μ*M + 5 *μ*M and tested for their combination effect in stimulating estrogenic activity. The results showed that, in two cotreatment conditions, the luciferase activity could be further increased as compared with that of single drug alone (Figure 1(c)). The relationship of drug-to-drug interaction was analyzed by the median-effect principle to calculate the possibility of synergism. Results showed that in estrogenic activity, the line of cotreatment (baicalein and daidzein) fell in the section of synergism in *F*_*a*_-CI plot (Chou-Talalay plot) (Figure 4(a)). In addition, the calculated CI value was smaller than 1 (i.e., 0.04587), and the two DRI values (92 for baicalein and 28.57 for daidzein) was higher than 1 (Figure 4(b)). These results clearly indicated that the combination of baicalein and daidzein could produce the synergistic effect in stimulating estrogenic activity in MCF-7 cells, and the favorable DRI (≫1) allowed a dose reduction that leaded to toxicity reduction in the therapeutic applications.

To further demonstrate the synergistic effect of two flavonoids, phosphorylation of ER was measured since this phosphorylation was required in transcriptional activation of ER. Experimentally, cultured MCF-7 cells were serum-starved and then treated with submaximal dose of baicalein (1 *μ*M) and daidzein (5 *μ*M) for 30 min. Phosphorylation of estrogen receptor *α* at serine 118 site (P-ER*α*; the classical site for estrogen-mediated ER activation) was detected by western blotting. Results showed that upon drug treatment, baicalein or daidzein could induce the receptor phosphorylation with different magnitudes. Here, daidzein showed better response in ER phosphorylation than of baicalein (Figure 1(d) upper panel). This receptor activation effects between baicalein and daidzein were rather consistent that diadzein was able to stimulate higher responses in luciferase activity and ER phosphorylation than that of baicalein.. Regarding the cotreatment of two flavonoids, the result expected was that the ER phosphorylation could be significantly potentiated when baicalein and daidzein were applied together (Figure 1(d) upper panel). Quantification of ER phosphorylation was performed to precisely indicate the fold of induction. At 30 min of drug treatment, the cotreatment of two flavonoids robustly induced the ER*α* phosphorylation up to 15-folds, which was significantly greater than that of the summation of baicalein (1-fold) and daidzein (2-folds) (Figure 1(d) lower panel). These results clearly demonstrated that, at submaximal concentration of baicalein and daidzein, the combination of two flavonoids was able to increase the estrogenic activity in terms of phosphorylation of ER*α* and transcriptional activation of ERE in MCF-7 cells.

**Figure 2:**
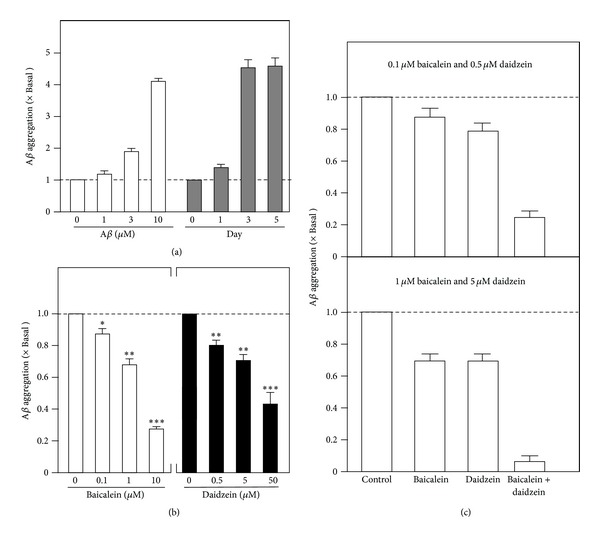
Combination of baicalein and daidzein enhances the *in vitro* anti-A*β* aggregation activity. (a) Different concentrations of unaggregated A*β* proteins (1, 3, and 10 *μ*M) were allowed to aggregate at 37°C for 3 days. Mixtures were measured by a fluorometric method using thioflavin binding assay. Besides, A*β* (10 *μ*M) was incubated at 37°C from 1 to 5 days and subjected to same fluorometric method to show the time-dependent aggregation activity. (b) Baicalein (0.1, 1, 10 *μ*M) or daidzein (0.5, 5, 50 *μ*M) was premixed with A*β* (10 *μ*M) protein before the aggregation at 37°C for 3 days to determine their anti-A*β* aggregation effect. (c) Baicalein and daidzein at 0.1 *μ*M + 0.5 *μ*M (upper panel) and 0.1 *μ*M + 0.5 *μ*M (lower panel) were combined together before the addition of A*β* protein for aggregation assay as in (b). Data are expressed as *x* Basal, whereas reading of control without drug is set as 1, mean ± SEM, *n* = 5. ***P < 0.001.

Estrogenic activity represents as one of the common biochemical properties of flavonoids. It is known that flavonoids possess different biological activities in different systems. Amongst all, neuroprotection related to Alzheimer's disease was chosen for analysis. Aggregation of A*β* is believed to be one of the cellular pathologies to cause the neuronal cell death in Alzheimer's disease. This A*β* aggregation could be measured in an *in vitro* condition. Monomeric A*β* at different concentrations were incubated at 37°C for 3 days to allow aggregation. The aggregated A*β* proteins were measured by a fluorometric method using thioflavin (ThT) as an indicator. Results showed that monomeric A*β*, from 1 to 10 *μ*M concentration, was aggregated to form polymers in a concentration-dependent manner (Figure 2(a)). In addition, this *in vitro* aggregation (at 10 *μ*M A*β*) was saturated after 4 days of incubation at 37°C. The formation of A*β* aggregate is required to increase the cytotoxicity of A*β* protein [36]. To investigate if flavonoids could reduce or inhibit the A*β* aggregation, baicalein or daidzein at different concentrations was mixed with 10 *μ*M A*β* and incubated for 3 days at 37°C. Results showed that the A*β* aggregation was found to be decreased by baicalein or daidzein in a dose-dependent manner (Figure 2(b)). The anti-A*β*-aggregation activity of daidzein was rather similar to that of baicalein. Interestingly, the A*β* aggregation could be further reduced by the cotreatment of two flavonoids at two testing concentrations (0.1 *μ*M + 0.5 *μ*M and 1 *μ*M + 5 *μ*M). At 0.1 *μ*M baicalein, the antiaggregation activity was ~10%, and that of 0.5 *μ*M daidzein was ~20%. In the cotreatment, the antiaggregation activity could be down to ~75%. Similar results were observed that at 1 *μ*M baicalein, the antiaggregation activity was ~34%, and that of 5 *μ*M diadzein was ~29%. In the cotreatment, the anti-aggregation activity could down to ~95% (Figure 2(c)). This synergistic effect of baicalein and daidzein was proven by the plot of *F*_*a*_-CI, the low CI value (0.01733), and high DRI values (85.51 for baicalein and 191.9 for daidzein) (Figure 4). These results were in agreement with those of estrogenic effects of two flavonoids; that is, the combination of baicalein or daidzein at low dosage could produce greater response.

**Figure 3:**
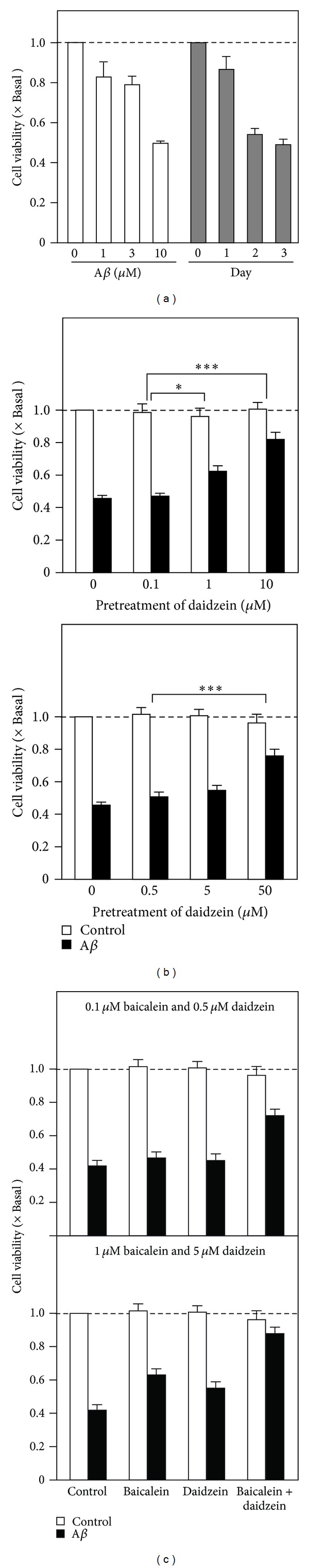
The neuroprotection effect of baicalein and daidzein against A*β*-induced cell death could be increased in the cotreatment. (a) Different concentrations of aggregated A*β* proteins (0, 1, 3, and 10 *μ*M) were added into PC12 cells for 48 hours, or A*β* (10 *μ*M) was added into the cultures for 1 to 3 days. Cell viability test using MTT colorimetric method was employed to determine the cytotoxicity of A*β*. (b) To determine the neuroprotection effect, PC12 cells were pretreated with baicalein (0.1, 1, 10 *μ*M; upper panel) or daidzein (0.5, 5, and 50 *μ*M; lower panel) for 24 hours before the addition of A*β* (10 *μ*M) for 3 days. (c) Baicalein and daidzein at 0.1 *μ*M + 0.5 *μ*M (upper panel) and 0.1 *μ*M + 0.5 *μ*M (lower panel) were mixed together and then treated with PC12 cells for 24 hours before the addition of A*β* protein for cytotoxicity assay as in (b). Data are expressed as *x* Basal, whereas reading of control without drug or time 0 min is set as 1, mean ± SEM, *n* = 5. ***P < 0.001.

**Figure 4:**
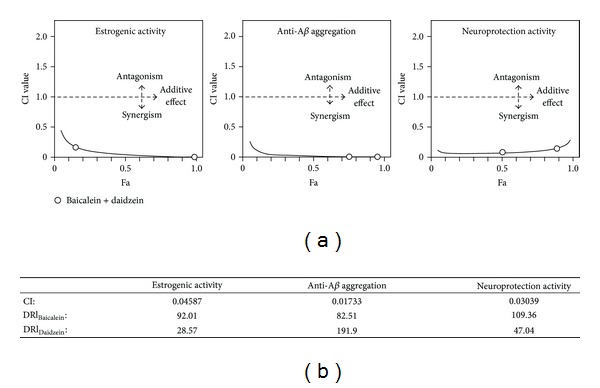
Analysis of synergism of baicalein and daidzein by median-effect principle. (a) Data from the estrogenic activity, *in vitro *anti-A*β* aggregation activity and neuroprotection activity assays were analyzed by the Chou-Talalay method as described in Section 2.2. The plots of Fa-CI in different assays were shown. (b) Data were also used to generate the CI and two DRI (for baicalein and daidzein) values.

In addition to the *in vitro* aggregation test, the cytotoxicity of A*β* attenuated by flavonoids would also be evaluated at cellular level. PC12 neuronal cells were chosen as a study model according to our previous experience [28]. To demonstrate the cytotoxicity of A*β* in PC12 cells, cultures were incubated with aggregated A*β* at different concentrations and different times and then subjected to cell viability assay. Results clearly indicated that the application of aggregated A*β* caused neuronal cell death in a dose-dependent manner and time-dependent manner (Figure 3(a)). This neuronal cytotoxicity of A*β* could be partially inhibited by the pretreatment of baicalein or daidzein (Figure 3(b)). Baicalein did not show any protective effect at 0.5 *μ*M and 5 *μ*M concentrations, and diadzein did not exert any effect at 0.1 *μ*M. On the hand, they could protect PC12 cells against A*β* at 10 *μ*M and 50 *μ*M concentrations, respectively. More importantly, the cotreatment of baicalein and daidzein at two testing doses (0.1 *μ*M + 0.5 *μ*M and 1 *μ*M + 5 *μ*M) could produce a significant neuroprotection activity as compared with that of individual flavonoid alone (Figure 3(c)). The cotreatment fell in the section of synergism in *F*_*a*_-CI plot (Figure 4). In addition, the CI value of cotreatment was 0.03039, while the DRI values of baicalein and daidzein were 109.36 and 47.04, respectively (Figure 4). All these results suggested that different flavonoids with less promising effects in neuroprotection could be greatly enhanced by the combination approach.

## 4. Discussion

Regarding the physiological roles of estrogen, the neuroprotective effects of estrogen in Alzheimer's and Parkinson's diseases have been widely documented; however, the molecular mechanisms are not fully understood [2, 10]. Owing to the adverse effects of estrogen in different clinical cases, including breast and ovarian cancers [37], the naturally occurring substances with estrogen-like activity can be a suitable substitute for estrogen for drug and health food development. In searching for substitutes of estrogen, flavonoids, a large group of compounds being naturally occurred in plants, vegetables, and herbal medicines, are commonly consumed in the forms of heath food supplement and/or food additive. They have been shown to possess a variety of biological effects in different systems. Different from estrogen, the administration of flavonoids could not initiate or induce cancer formation [3, 15]. In another study, a high concentration of phytoestrogen could even significantly inhibit the proliferation of cancer cell growth [18], which suggests the safe consumption of flavonoids.

According to our previous results in screening the biological activities of flavonoids [27, 28, 30]. most flavonoids from the subclass of isoflavones have the estrogenic property; that is, they can stimulate phosphorylation estrogen receptor *α* and induce the transcriptional activity of estrogen responsive element in MCF-7 breast cells. In contrast, the estrogenic activities in other subclasses are rather diverse. Therefore, it is hard to have a conclusion on the structure-function relationship of these flavonoids, even though most flavonoids share a similar core structure. For example, Radix Notoginseng flavonol glycoside (RNFG), a flavonoid isolated from the roots of *Panax notoginseng *with estrogenic activity, could protect neurons against A*β*-induced cytotoxicity. Interestingly, another two flavonoids called quercetin and rutin, with similar estrogenic activity and structure to RNFG, did not show any neuroprotection effect in neurons [38]. In our results, an interesting pheromone was observed which is treatment of baicalein (10 *μ*M) and diadzein (50 *μ*M) could produce higher responses of inducing ERE activity than those of estrogen (at 10 nM) in transfected MCF-7 cells. We speculated that the binding complex of flavonoid and ER*α* (and/or ER*β*) might be more stable and exerted the higher transcriptional response than that of estrogen. However, further investigation is required for validation.

Estrogen receptors could be involved in estrogen- or flavonoid-mediated neuroprotection [2, 39]. Interestingly, the neuroprotective effect of the flavonoids cannot be fully explained by the estrogenic effect or which is not the only factor involved in neuroprotection [40, 41]. Based on the current study, baicalein and daidzein are the two effectors in activating estrogen-mediated pathway and preventing A*β*-induced neuronal death. Baicalein is the major ingredient of a commonly used Chinese medicine-Radix Scutellariae (roots of *Scutellaria baicalensis*). On one hand, Radix Scutellariae is being used as an anti-inflammation agent clinically, and anti-inflammation agents have been proposed to treat neurodegenerative diseases [42]. On the other hand, baicalein has been shown to exert neuroprotective effects against glutamate/NMDA stimulation, glucose deprivation, oxidative stress, A*β*-induced toxicity, and inflammation-mediated degeneration [43, 44]. Daidzein is mainly from soybean extracts and considered as phytoestrogen used clinically. With the hints of neuroprotection of estrogen, daidzein and soybean extracts can improve memory and have neuroprotective roles in brain [14, 15]. The previously stated biological activities and clinical applications of the herbal medicines further confirm our results.

One of the ultimate goals of pharmacotherapy, using a combination of different drugs with similar biological effects, is to increase the therapeutic efficacy of treatment and decrease the drug toxicity. Multidrug therapy has been applied in medical areas such as hypertension, AIDS, antibiotic therapy, and neurodegenerative diseases for a period of time. This combination therapy is consistent with the theory of traditional Chinese medicines that a mixture of herb can produce a harmonic and synergistic actions to the target(s). Here, the synergy of baicalein and daidzein in stimulating estrogenic effects could be explained, for example, by another pathway (nonestrogen receptor-mediated signaling pathway) [45] or different binding specificity of estrogen receptors subtypes to baicalein and daidzein [46]. In addition, daidzein can potentiate the neuroprotection of baicalein, which may be resulted from different mechanisms of their neuroprotection or pharmacokinetic interactions of one flavonoid in altering the solubility, absorption, distribution, metabolism or elimination of the other [47]. Such potentiation effects were produced by using a submaximal or minimal concentration, which acted as an effective way to enhance the biological effects of different flavonoids. Otherwise, side effects may be observed when high working concentration of flavonoids is used. 

## 5. Conclusion

In this study, we employed a combination approach for efficacy optimization. By combining the appropriate flavonoids, daidzein and baicalein in our case, their cellular and biological activities could be maximized which would be useful for developing health supplements and/or therapeutic drugs against Alzheimer's diseases in future.
